# Die Schwangere mit COVID-19-ARDS auf der Intensivstation

**DOI:** 10.1007/s00101-024-01405-5

**Published:** 2024-04-26

**Authors:** J. Kalbhenn, O. Marx, K. Müller-Peltzer, M. Kunze, H. Bürkle, J. Bansbach

**Affiliations:** 1 Klinik für Anästhesiologie und Intensivmedizin, Universitätsklinik Freiburg, Hugstetter Str. 55, Freiburg, Deutschland; 2 Klinik für Radiologie, Universitätsklinik Freiburg, Freiburg, Deutschland; 3 Klinik für Frauenheilkunde, Geburtshilfe und Perinatologie, Universitätsklinik Freiburg, Freiburg, Deutschland

**Keywords:** Schwangerschaft, COVID-19, Bauchlagerung, ARDS, ECMO, Pregnancy, COVID-19, Prone positioning, ARDS, ECMO

## Abstract

**Hintergrund:**

Schwangere mit einer SARS-CoV-2-Infektion (COVID-19) haben ein erhöhtes Risiko für einen schweren Verlauf der COVID-19. Die medizinische und ethische Abwägung maternaler und fetaler Risiken und die Priorisierung von Therapieoptionen stellen eine große Herausforderung auf der Intensivstation dar. Eine enge interdisziplinäre Abstimmung ist unabdingbar.

**Ziel der Arbeit:**

Beschreibung und Diskussion intensivmedizinischer Behandlungsstrategien und des perinatalen anästhesiologischen Managements bei Patientinnen mit COVID-19-ARDS (CARDS).

**Material und Methoden:**

Analyse von demografischen Daten, Anamnese, klinischem Management, Komplikationen, Indikationen und Management der extrakorporalen Membranoxygenierung (ECMO) sowie des kindlichen Überlebens aller schwangeren Patientinnen, die zwischen März und November 2021 auf der anästhesiologischen Intensivstation eines deutschen Universitätsklinikums wegen eines schweren CARDS behandelt wurden.

**Ergebnisse:**

Kohorte von 9 konsekutiven Patientinnen mit einem Durchschnittsalter von 30,3 Jahren (Min–Max: 26 bis 40 Jahre) und einem Schwangerschaftsalter von 21 + 3 bis 37 + 2 Wochen. Keine der Patientinnen war gegen SARS-CoV‑2 geimpft. Zwei Patientinnen mussten mit inhalativem Stickstoffmonoxid und venovenöser ECMO behandelt werden. Alle Frauen und 5 Neugeborene haben überlebt. Zwei Patientinnen wurden mit intakter Schwangerschaft nach Hause entlassen. Alle Kinder wurden durch einen Kaiserschnitt entbunden. Es wurden 2 intrauterine fetale Todesfälle beobachtet. Keines der Neugeborenen wurde bei der Geburt positiv auf SARS-CoV‑2 getestet.

**Diskussion:**

Das peripartale Management erfordert eine enge interdisziplinäre Zusammenarbeit und sollte in der Frühschwangerschaft vorrangig auf das mütterliche Überleben ausgerichtet sein. Die Bauchlagerung, ein wesentlicher, evidenzbasierter Eckpfeiler in der Therapie des akuten Atemnotsyndroms (ARDS), kann auch in fortgeschrittenen Schwangerschaftsstadien sicher angewendet werden. Inhalatives Stickstoffmonoxid (iNO) und extrakorporale Membranoxygenierung (ECMO) sollten als lebensrettende Behandlungsoptionen für sorgfältig ausgewählte Patientinnen in Betracht gezogen werden.

## Abstract (en)

**Background:**

Pregnant women with coronavirus disease 2019 (COVID-19) are at increased risk of severe disease progression. Comorbidities, such as chronic arterial hypertension, diabetes mellitus, advanced maternal age and high body mass index, may predispose to severe disease. The management of pregnant COVID-19 patients on the intensive care unit (ICU) is challenging and requires careful consideration of maternal, fetal and ethical issues.

**Objective:**

Description and discussion of intensive care treatment strategies and perinatal anesthesiological management in patients with COVID-19 acute respiratory distress syndrome (CARDS).

**Material and methods:**

We analyzed the demographic data, maternal medical history, clinical intensive care management, complications, indications and management of extracorporeal membrane oxygenation (ECMO) and infant survival of all pregnant patients treated for severe CARDS in the anesthesiological ICU of a German university hospital between March and November 2021.

**Results:**

The cohort included 9 patients with a mean age of 30.3 years (range 26–40 years). The gestational age ranged from 21 + 3 weeks to 37 + 2 weeks. None of the patients had been vaccinated against SARS-CoV‑2. Of the nine patients seven were immigrants and communication was hampered by inadequate Central European language skills. Of the patients five had a P_a_O_2_/F_i_O_2_ index < 150 mm Hg despite escalated invasive ventilation (F_i_O_2_ > 0.9 and a positive end-expiratory pressure [PEEP] of 14 mbar) and were therefore treated with repeated prolonged prone positioning maneuvers (5–14 prone positions for 16 h each, a total of 47 prone positioning treatments) and 2 required treatment with inhaled nitric oxide and venovenous ECMO. The most common complications were bacterial superinfection of the lungs, urinary tract infection and delirium. All the women and five neonates survived. All newborns were delivered by cesarean section, two patients were discharged home with an intact pregnancy and two intrauterine fetal deaths were observed. None of the newborns tested positive for SARS-CoV‑2 at birth.

**Conclusion:**

High survival rates are possible in pregnant patients with CARDS. The peripartum management of pregnant women with CARDS requires close interdisciplinary collaboration and should prioritize maternal survival in early pregnancy. In our experience, prolonged prone positioning, an essential evidence-based cornerstone in the treatment of ARDS, can also be safely used in advanced stages of pregnancy. Inhaled nitric oxide (iNO) and ECMO should be considered as life-saving treatment options for carefully selected patients. For cesarean section, neuraxial anesthesia can be safely performed in patients with mild CARDS if well planned but the therapeutic anticoagulation recommended for COVID-19 may increase the risk of bleeding complications, making general anesthesia a more viable alternative, especially in severe disease.

Auch wenn die COVID-19-Pandemie (Coronavirus-Krankheit-2019) überwunden scheint, können aus der retrospektiven Betrachtung der Erfahrungen bei der Behandlung kritisch kranker Schwangerer wichtige Schlussfolgerungen für die zukünftige Therapie des Lungenversagens bei dieser Patientinnengruppe abgeleitet werden.

## Hintergrund

Mit mehr als 750 Mio. Infizierten und mehr als 6,8 Mio. Todesfällen weltweit seit Dezember 2019 war und ist die Coronavirus-Erkrankung (COVID-19) für eine globale Krise der öffentlichen Gesundheit verantwortlich [1, 2]. Schwangere Frauen gelten in diesem Zusammenhang als Hochrisikogruppe, da physiologische Veränderungen des kardiopulmonalen Systems während der Schwangerschaft, wie Zwerchfellhochstand mit verminderter funktioneller Residualkapazität, erhöhter Sauerstoffverbrauch und Atemwegsödeme die Entwicklung eines akuten Atemnotsyndroms des Erwachsenen (ARDS) bei COVID-19 begünstigen [2].

Schwangere Frauen mit COVID-19 weisen im Vergleich zu Nichtschwangeren eine höhere Rate an Krankenhausaufenthalten und Aufnahmen auf der Intensivstation auf und benötigten während der Intensivbehandlung häufiger eine mechanische Beatmung [3]. Darüber hinaus können vorbestehende Komorbiditäten wie chronische arterielle Hypertonie, Diabetes mellitus, hohes mütterliches Alter und hoher Body-Mass-Index zu einem schweren Krankheitsverlauf beitragen [4]. Mütterliches Atem- und Nierenversagen sowie thromboembolische Ereignisse bedrohen sowohl die Mutter als auch das Ungeborene und können eine spezifische Therapie auf der Intensivstation erforderlich machen.

Die genauen Auswirkungen einer maternalen SARS-CoV-2-Infektion auf den Fetus sind noch nicht vollständig geklärt. Einige Studien bei Schwangeren mit COVID-19 zeigen eine höhere Wahrscheinlichkeit für eine Frühgeburt im Vergleich zu Schwangeren ohne COVID-19 sowie eine höhere Wahrscheinlichkeit für die Notwendigkeit einer postpartalen neonatologischen Intensivbehandlung [4, 5]. Andere observative Studien legen nahe, dass COVID-19 nicht mit einem erhöhten Risiko für das Neugeborene verbunden ist, bzw. für eine Aussage dazu mehr qualitativ hochwertige Daten notwendig sind [6]. Wei et al. schätzen, dass COVID-19 das Risiko eines intrauterinen Fruchttodes oder einer Totgeburt um mehr als das Doppelte erhöht [7]. Da eine schwere COVID-19-Erkrankung einer Form der thrombotischen Mikroangiopathie entspricht [8] ist eine thrombotische Beteiligung der Plazenta mit konsekutiver fetaler Minderversorgung denkbar.

Die Bauchlagerungstherapie ist ein wesentlicher Bestandteil der Behandlung des ARDS. Die Lagerungstherapie widerspricht jedoch den pathophysiologischen Annahmen und Konzepten zur Vermeidung eines Vena-cava-Kompressionssyndroms bei Schwangeren, sodass bei der Durchführung der Bauchlagerung darauf geachtet werden sollte, dass der Uterus in der Bauchlage die V. cava nicht komprimiert.

Die coronavirusinduzierte Koagulopathie per se ist vergesellschaftet mit schweren thromboembolischen Komplikationen. In Verbindung mit dem prokoagulatorischen Zustand in der Schwangerschaft kann eine pharmakologische Antikoagulation sinnvoll sein, gleichzeitig aber auch das Risiko für die Entstehung eines Plazentahämatoms und einer peripartalen Blutung erhöhen.

Diese Fallserie beschreibt unser therapeutisches Vorgehen bei schwangeren und peripartalen Frauen mit COVID-19-assoziiertem ARDS, gefolgt von einer kritischen Diskussion klinischer Behandlungsstrategien und des peripartalen anästhesiologischen Managements.

## Studiendesign

Wir identifizierten alle schwangeren und peripartalen Patientinnen mit einer bestätigten SARS-CoV-2-Infektion, die in unserem nach internationalen Empfehlungen [8] ausgestatteten und von der Deutschen Gesellschaft für Anästhesiologie und Intensivmedizin (DGAI) zertifizierten deutschen universitären ARDS-Zentrum zwischen März und November 2021 intensivmedizinisch behandelt wurden (vorherrschende Coronavariante: Delta B 1.617.2). Wir analysierten demografische Daten, mütterliche Anamnese, allgemeines klinisches Management, Komplikationen, die spezifische Behandlung des Coronavirus-induzierten akuten Atemnotsyndroms (CARDS), Indikationen, Management und Besonderheiten der extrakorporalen Membranoxygenierung (ECMO) sowie mütterliches und kindliches Überleben. Sämtliche Patientendaten wurden anonymisiert erfasst. Die Analyse wurde von der lokalen Ethikkommission (EK 235/20) begutachtet und genehmigt. Aufgrund des deskriptiven, nichtinterventionellen und anonymen Designs der Studie und der besonderen Umstände, während der COVID-19-Pandemie konnte auf die Einholung einer schriftlichen Einverständniserklärung verzichtet werden. Die Studie wurde in Übereinstimmung mit der Initiative zur Verbesserung der Berichterstattung über epidemiologische Beobachtungsstudien (STROBE) unter Verwendung der vorgeschlagenen Checkliste für epidemiologische Kohortenstudien durchgeführt.

## Ergebnisse

### Patientinnen und Vorgeschichte

Unsere Kohorte umfasst 9 Patientinnen mit einem Durchschnittsalter von 30,3 Jahren (Min–Max: 26 bis 40 Jahre). Acht Patientinnen waren zum Zeitpunkt der Aufnahme auf die Intensivstation schwanger (21 + 3 bis 32 + 4 Schwangerschaftswoche), eine weitere war unmittelbar postpartal (37 + 2 Schwangerschaftswoche). Sieben der 9 Patientinnen hatten einen Migrationshintergrund, und die Erhebung einer ausführlichen Anamnese mit ihnen und ihren Angehörigen war sprachlich erschwert. Mutterpässe und Vorsorgeuntersuchungen waren lückenhaft und unvollständig. Soweit wir dies unter den gegebenen Umständen nachvollziehen konnten, litten vor der Hospitalisierung 2 Patientinnen während der Schwangerschaft an einem Harnwegsinfekt, und eine Patientin entwickelte einen milden Schwangerschaftsdiabetes. Ansonsten verliefen die Schwangerschaften komplikationslos. Keine der Patientinnen war gegen SARS-CoV‑2 geimpft worden. Fünf der 9 Patientinnen waren adipös (2 Patientinnen mit Adipositas Grad I, 3 Patientinnen mit Adipositas Grad II).

### Kortikoidtherapie

Entsprechend den Empfehlungen der zum Behandlungszeitraum gültigen S3-Leitlinie zur stationären Therapie von Patientinnen mit COVID-19 [9] erhielten 5 Patientinnen 6 mg Dexamethason für insgesamt 10 Tage. Zwei Patientinnen waren bereits vor der Verlegung in unsere Klinik mit Prednisolon bzw. mit Methylprednisolon behandelt worden. Zwei Patientinnen erhielten kein Kortikosteroid, eine aufgrund eines eher milden Verlaufs, die andere, weil der Symptombeginn mehr als 10 Tage zurücklag. Beide Entscheidungen wurden auf der Grundlage von inzwischen überarbeiteten klinikinternen Empfehlungen zum jeweiligen Behandlungszeitpunkt getroffen.

### CARDS-Therapie

#### Schweres CARDS: p_a_O_2_/F_I_O_2_ ≤ 100 mm Hg

Sechs Patientinnen entwickelten ein schweres CARDS mit einem medianen p_a_O_2_/F_I_O_2_-Index von 71 mm Hg (niedrigster p_a_O_2_/F_I_O_2_-Index, während der Intensivbehandlung: 43–92 mm Hg), das bei 5 Patientinnen eine invasive mechanische Beatmung erforderlich machte. Eine Patientin zeigte unter nichtinvasiver Beatmungstherapie (NIV) eine rasche Verbesserung des pulmonalen Gasaustausches und musste nicht endotracheal intubiert werden. Fünf dieser 6 Patientinnen wiesen trotz eskalierter invasiver Beatmung (F_I_O_2_ > 0,9 und PEEP von 14 mbar) einen p_a_O_2_/F_I_O_2_-Index < 150 mm Hg auf und wurden daher mit prolongierten Bauchlagerungstherapien (5 bis 14 Bauchlagerungen à 16 h, insgesamt 47 Lagerungstherapien à 16 h) behandelt. Wegen therapierefraktärer Hypoxie trotz eskalierter invasiver Beatmung- und Lagerungstherapie wurden 2 von ihnen zusätzlich mit inhalativem Stickstoffmonoxid (iNO) behandelt (Patientin 1: p_a_O_2_/F_I_O_2_-Index zu Beginn der NO-Therapie: 64 mm Hg, NO 40 ppm über 16 h; Patientin 6: p_a_O_2_/F_I_O_2_-Index zu Beginn der NO-Therapie: 66 mm Hg, NO 40 ppm über 31 h).

Bei beiden Patientinnen konnte durch die Gabe von inhalativem NO keine Verbesserung der Oxygenierung erreicht werden, sodass beide Patientinnen mit einer venovenösen ECMO-Therapie behandelt werden mussten (Patientin 1: p_a_O_2_/F_I_O_2_-Index: 61 mm Hg, vv-ECMO für 12 Tage, doppellumige Kanüle jugulär, maximaler Pumpenfluss 5,1 l/min, maximaler Gasfluss 6,0 l/min; Patientin 6: p_a_O_2_/F_I_O_2_-Index: 43 mm Hg, vv-ECMO für 15 Tage, femorojugulär, maximaler Pumpenfluss 7,2 l/min, maximaler Gasfluss 9 l/min).

#### Moderates CARDS: p_a_O_2_/F_I_O_2_ = 101–200 mm Hg

Zwei Patientinnen wiesen bei Aufnahme auf die Intensivtherapiestation ein moderates CARDS mit einem p_a_O_2_/F_I_O_2_-Index von 110 mm Hg bzw. von 178 mm Hg auf und wurden mit nichtinvasiver Beatmung und intensivierter Atem- und Physiotherapie behandelt.

#### Mildes CARDS: p_a_O_2_/F_I_O_2_ = 201–300 mm Hg

Eine Patientin zeigte bei Aufnahme auf die Intensivtherapiestation ein leichtes CARDS mit einem p_a_O_2_/F_I_O_2_-Index von 282 mm Hg, das mit nasaler High-Flow-Sauerstofftherapie sowie Atem- und Physiotherapie behandelt werden konnte (Tab. 1 und 2).

**Tab. 1:** Demografische Daten, COVID-19-assoziierte Symptome und Therapien, Komplikationen, Beatmungsparameter und geburtsspezifische Daten der Patientinnen

	Patientin 1	Patientin 2	Patientin 3	Patientin 4	Patientin 5	Patientin 6	Patientin 7	Patientin 8	Patientin 9
**Mütterliche Faktoren**									
*Alter (Jahre)*	29	40	26	26	29	33	27	32	31
Herkunft	Arabisch	Asiatisch	Arabisch	Kaukasisch	Arabisch	Persisch	Kaukasisch	Asiatisch	Arabisch
*BMI (kg/m²)*	31,3	22,8	35,7	35,1	36,1	32,65	22,3	20,7	25,4
*Komorbiditäten*	Adipositas	Hepatitis B	Adipositas	Adipositas	Adipositas	Adipositas	Paranoide Schizophrenie	Keine	Keine
*Schwangerschaftsbed. Komorbiditäten* Impfstatus SARS-CoV	Keine Negativ	Keine Negativ	Harnwegsinfekt Negativ	Keine Negativ	Keine Negativ	Schwangerschaftsdiabetes Negativ	Harnwegsinfekt Negativ	Keine Negativ	Keine Negativ
**COVID-19 der Mutter**									
*Auftreten von Symptomen bis zum positiven Test (Tage)*	4	4	2	5	4	4	4	4	1
*Krankenhausaufenthalt (Tage) vor* ITS Zuverlegung von externem Krankenhaus (Tag nach Hospitalisation)	6 Ja (2)	12 Ja (13)	4 Nein	7 Nein	8 Nein	9 Ja (4)	4 Ja (5)	7 Ja (3)	1 Ja (3)
*Endotracheale Intubation* (Tag nach KH-Aufnahme)	6	13	#	#	#	11	#	10	3
*Virusvariante*	B 1.1.7	#	#	B 1.1.7	B 1.617.2	B 1.617.2	B 1.617.2	B 1.617.2	B 1.617.2
*COVID-19-Therapie*	Dexamethason	Keine	Dexamethason	Keine	Dexamethason	Prednisolon	Dexamethason	Dexamethason	Methylprednisolon, ASS
*Bakterielle Superinfektion während der Behandlung* *Laborwerte bei Aufnahme auf die ITS*	Ja	Ja	Ja	Nein	Nein	Ja	Ja	Ja	Nein
*Leukozytenzahl (10*^*3*^*/μl)*	10,41	19,34	7,18	8,72	4,03	10,64	7,7	10,1	12,0
*Prokalzitonin (ng/ml)*	0,43	2,68	< 0,05	#	0,07	1,41	0,44	3,05	0,82
*D‑Dimer (mg/l)*	2,3	4	0,88	#	1,07	1,23	1,34	0,99	0,8
*Fibrinogen (mg/dl)* *Komplikationen:* *Akutes Nierenversagen* *Hämodialyse* (Tage)	436 Urosepsis, HSV-Infektion, Mastitis Ja Nein	566 Delir Nein	# Harnwegsinfekt Nein	602 # Nein	449 # Nein	621 Delir, Perikardtamponade, VHF, Harnwegsinfekt Ja Ja	493 # Nein	479 Atone Nachblutung Nein	383 Delir, Harnwegsinfekt Nein
*Nichtinvasive Beatmung (Tage)*	Ja (1)	Ja (7)	Ja (17)	Nein	Ja (7)	Ja (1)	Ja (6)	Ja (2)	Ja (5)
*Invasive Beatmung (Tage)*	Ja (42)	Ja (12)	Nein	Nein	Nein	Ja (38)	Nein	Ja (7)	Ja (7)
*PEEP/Plateau (mbar)*	12/29	14/20	#	#	#	14/33	#	14/22	14/30
*Niedrigster p*_*a*_*O*_*2*_*/*F_I_O_2_*-Index (mm* *Hg)*	61	92	110	282	178	43	79	81	69
*NO-Therapie (h)*	Ja (16)	Nein	Nein	Nein	Nein	Ja (31)	Nein	Nein	Nein
*Lagerungstherapie*	Ja (14)	Ja (9)	Nein	Nein	Nein	Ja (12)	Nein	Ja (5)	Ja (7)
*ECMO (t)* *Tracheotomie*	Ja (12) Ja	Nein Nein	Nein Nein	Nein Nein	Nein Nein	Ja (15) Ja	Nein Nein	Nein Nein	Nein Nein
*Schwangerschaft*	II/I	III/I	II/I	III/II	IV/II	IV/III	II/I	I/0	IV/II
*Gestationsalter bei der Aufnahme auf die ITS* Gestationsalter bei Entlassung aus dem Krankenhaus Auslösender Faktor für Geburt	24 + 1	29 + 3 Pathologisches CTG	29 + 4 Beginnende Wehen	Postpartal Beginnende Wehen	24 + 5 26 + 2	21 + 3	30 + 3 32 + 1	30 + 2 Pathologisches CTG	32 + 4 Beginnende Wehen
Geburt	#	32 + 0	32 + 2	37 + 2	–	#	–	31 + 0	33 + 1
*Geburtsgewicht (g)*	–	1650	1710	3175	–	–	–	1320	2170
*APGAR*	–	4/6/10	4/7/10	10/10/10	–	–	–	4/5/9	4/6/10
*SARS-CoV-2-PCR (Ct) Neugeborene*	–	Negativ	Negativ	Negativ	–	–	–	Negativ	Negativ

*#* Merkmal nicht vorhanden

**Tab. 2:** Computertomographieaufnahmen des Thorax zum Zeitpunkt des Behandlungsbeginns auf der Intensivtherapiestation sowie, soweit diese durchgeführt wurden, Thoraxröntgenaufnahmen im Verlauf der intensivmedizinischen Behandlung

	Computertomographie des Thorax bei Beginn der Behandlung	Röntgenaufnahme des Thorax x Tage nach Beginn der Behandlung
Pat. 1	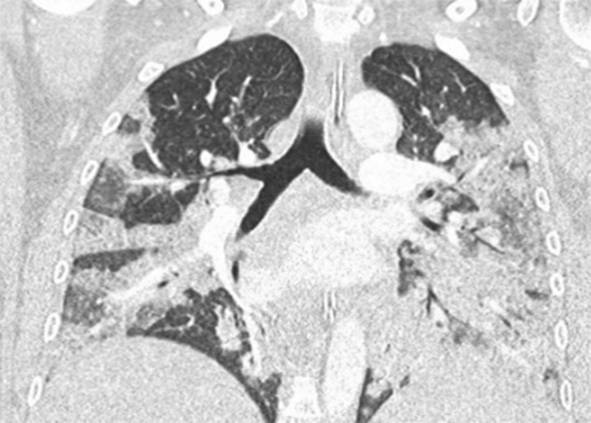	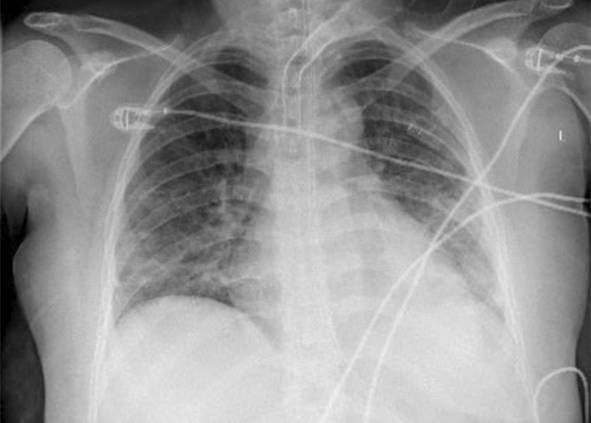
		+40 Tage
Pat. 2	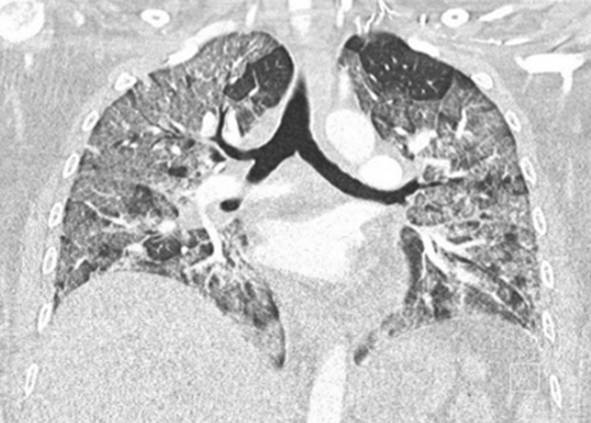	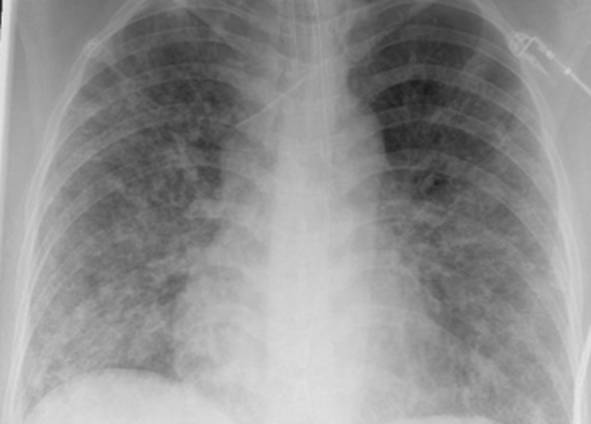
		+11 Tage
Pat. 3	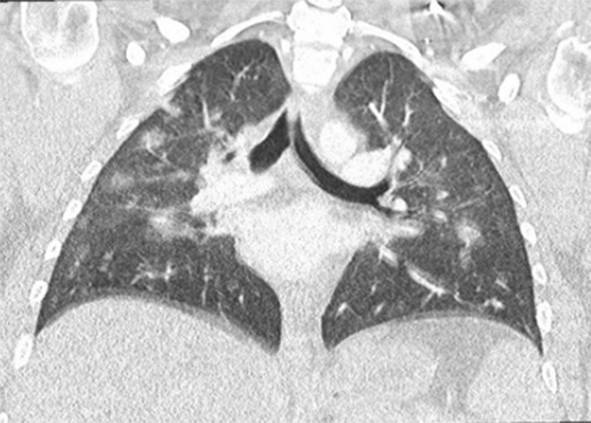	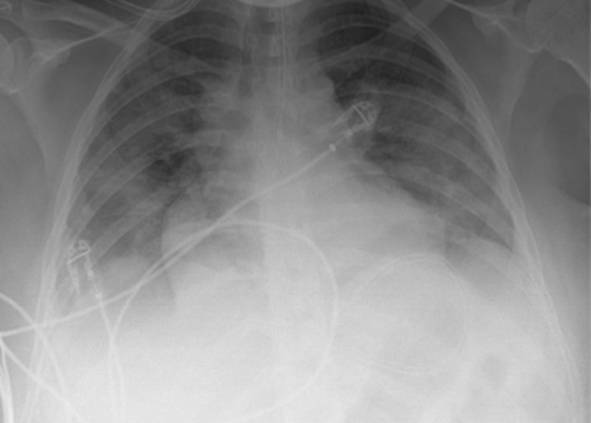
		+5 Tage
Pat. 4	/	/
Pat. 5	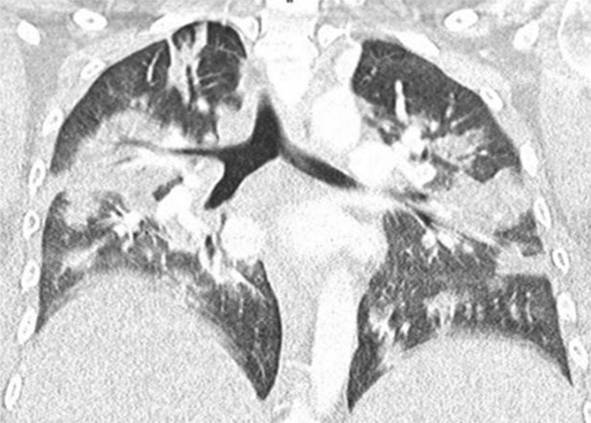	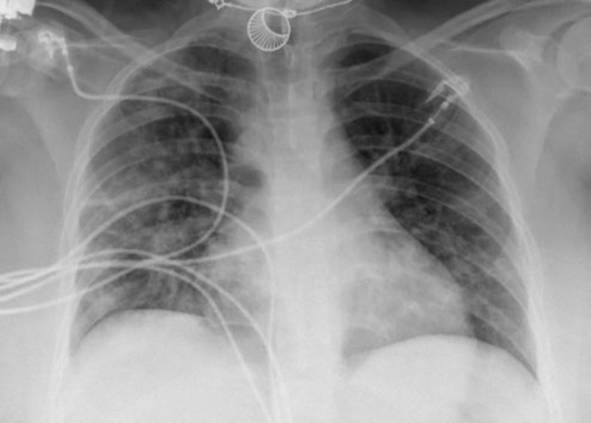
		+5 Tage
Pat. 6	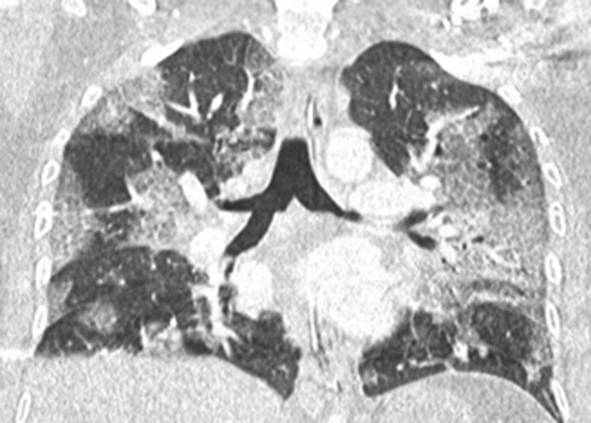	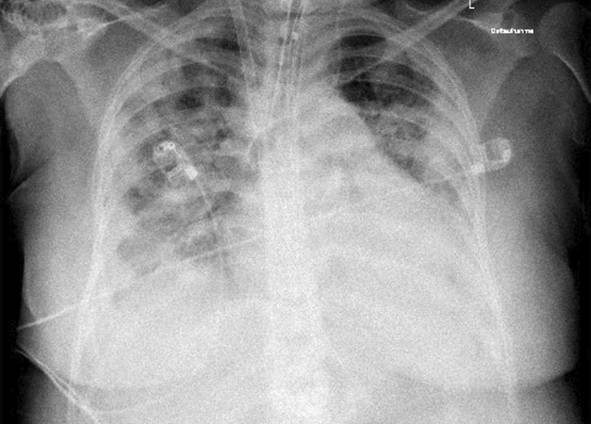
		+14 Tage
Pat. 7	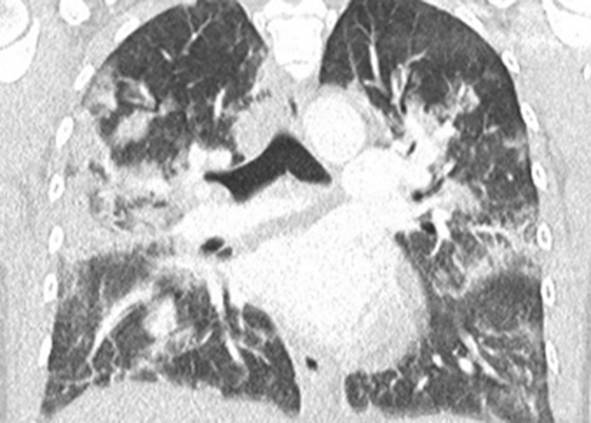	/
Pat. 8	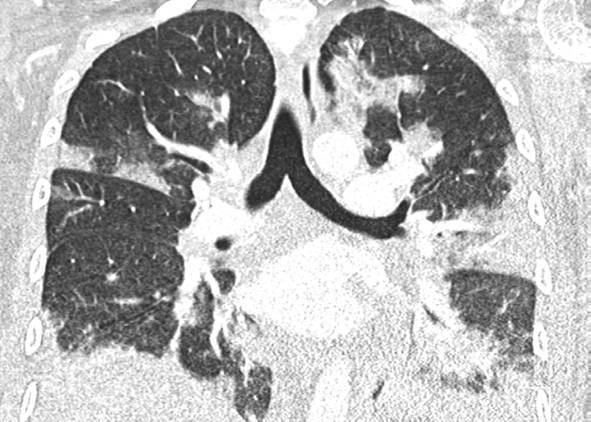	/
Pat. 9	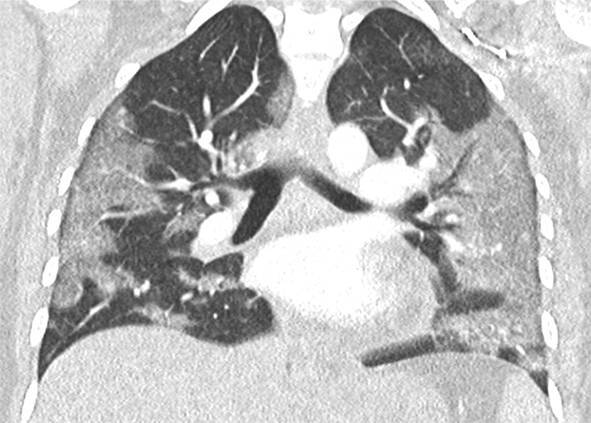	/

### Mütterliche Komplikationen und kindliches Outcome

Die häufigsten mütterlichen Komplikationen waren bakterielle Superinfektionen der Lunge, Harnwegsinfektionen und Delir (Tab. 1). Fünf Kinder wurden erfolgreich per Kaiserschnitt entbunden, 2 Patientinnen wurden mit intakter Schwangerschaft nach Hause entlassen, und 2 Patientinnen erlitten einen intrauterinen Fruchttod aufgrund einer Plazentathrombose mit anschließender vaginaler Totgeburt. Die Indikation zur Sectio caesarea wurde interdisziplinär gestellt. Dabei wurden Faktoren wie z. B. der Krankheitsverlauf der Mutter, das Schwangerschaftsalter, ein pathologisches Kardiotokogramm (CTG) oder einsetzende Wehen berücksichtigt. Keines der Neugeborenen wurde bei der Geburt positiv auf SARS-CoV‑2 getestet. Vier der 5 Neugeborenen wurden vor dem errechneten Geburtstermin entbunden und wiesen ein niedriges Geburtsgewicht auf (Tab. 1).

## Diskussion

Die besondere Herausforderung für die Behandler schwangerer Patientinnen besteht darin, für 2 Lebewesen, die unterschiedliche und möglicherweise gegensätzliche Therapien benötigen, gleichzeitig Sorge tragen zu müssen. Die Behandlungsprioritäten können sich im Verlauf der Schwangerschaft ändern: Steht zu Beginn der Schwangerschaft das Überleben der Mutter im Vordergrund, so rückt mit zunehmender Überlebenswahrscheinlichkeit des ungeborenen Kindes das gemeinsame Wohl beider in den Mittelpunkt der therapeutischen Bemühungen. Eine pragmatische Einteilung in Behandlungsgruppen abhängig von der Schwangerschaftsdauer kann dem Kliniker helfen, die lebensrettende Behandlung zu strukturieren und Prioritäten zu setzen. Eine solche Einteilung birgt aber auch die Gefahr, der einzelnen Schwangeren bzw. ihrem Kind individuell nicht vollständig gerecht zu werden.

Auf der Grundlage unserer Erfahrungen haben wir für uns 3 verschiedene Gruppen von schwangeren COVID-19-Patientinnen mit CARDS identifiziert, für die wir jeweils im Detail unterschiedliche Behandlungsprioritäten vorschlagen:

Gruppe I: Schwangere Patientinnen kurz vor dem errechneten Geburtstermin (Gestationsalter 34 + 0–36 + 0 SSW): Um eine konsequente Versorgung der Mutter, einschließlich spezifischer pharmakologischer Behandlung mit potenziell embryotoxischen Substanzen und Bauchlagerungstherapie zu ermöglichen, haben wir im interdisziplinären Konsens eine zeitnahe Entbindung per Kaiserschnitt angestrebt. Kritische Abwägung und enge interdisziplinäre Zusammenarbeit sollten einer solchen Entscheidung stets vorangehen [10, 11].

Ob eine Entbindung tatsächlich zu einer Verbesserung der mütterlichen respiratorischen Insuffizienz führt, ist unklar [12]. In unserer Erfahrung hat sie jedoch insbesondere die Lagerungstherapie erleichtert.

Gruppe II: Bei einem Gestationsalter von 24 + 0–33 + 6 SSW ist eine grundsätzliche Lebensfähigkeit des Kindes gegeben. Mit jeder zusätzlichen Woche sinken die neonatale Morbidität und Mortalität signifikant [13, 14], weshalb eine Prolongation der Schwangerschaft aus fetaler Sicht anzustreben ist. Aufgrund des erhöhten Sauerstoffbedarfs des mütterlichen und fetalen Gewebes muss die Atemarbeit der Mutter während der Schwangerschaft erhöht werden. Bei Schwangeren mit schwerem CARDS ist eine solche Anpassung jedoch nur schwer möglich, sodass aus ausschließlich maternaler Sicht die Beendigung des Zustandes des erhöhten Sauerstoffbedarfs – also die Termination der Schwangerschaft – lebensrettend sein kann. Bei diesen Patientinnen konzentrierte sich die Therapie auf das mütterliche Überleben, einschließlich Bauchlagerung, Behandlung mit inhalativem Stickstoffmonoxid und ggf. ECMO-Therapie. Die Fortsetzung der Schwangerschaft stellte ein simultanes Behandlungsziel dar, wenn die Aufrechterhaltung der Schwangerschaft die notwendige Behandlung der Mutter nicht unmöglich machte. In diesen Fällen wurde eine vorzeitige Entbindung täglich interdisziplinär diskutiert und ggf. indiziert. Da die Patientinnen mit schwerem CARDS nicht in der Lage waren, Entscheidungen für sich und ihr ungeborenes Kind zu treffen, haben wir neben den beteiligten Fachdisziplinen nach Möglichkeit auch die Angehörigen in diese komplexen Entscheidungsprozesse einbezogen.

Gruppe III: Bei einer Schwangerschaft vor dem lebensfähigen Stadium des Fetus (Gestationsalter < (22 + 0–)24 + 0 SSW) sollte sich die Therapie schwerpunktmäßig auf die Behandlung der Mutter konzentrieren [15]. Die Grenzen des kindlichen Überlebens haben sich in den letzten Jahren immer weiter nach unten verschoben [16, 17]. Eine alleinige Priorisierung auf das mütterliche Wohl könnte sich daher in Zukunft auch in dieser Gruppe relativieren.

### Risikofaktoren für einen schweren Verlauf von COVID-19 bei Schwangeren

Die Risikofaktoren für einen schweren Verlauf von COVID-19 bei schwangeren Patientinnen wurden in zahlreichen Studien untersucht. Vouga et al. identifizierten vorbestehende Lungenerkrankungen, kardiovaskuläre Erkrankungen, arterielle Hypertonie und Diabetes mellitus als Risikofaktoren für einen komplikativen Verlauf. Für Adipositas (BMI > 30) und höheres Alter der Schwangeren (> 35 Jahre) konnte kein Zusammenhang mit einem schweren Verlauf nachgewiesen werden [18]. Neuere Daten bestätigen die oben genannten Risikofaktoren, fanden aber auch einen Zusammenhang mit Adipositas (BMI > 30) und Untergewicht (BMI < 18,5) [19]. In unserer Kohorte litten 5 von 9 Patientinnen an Adipositas. Sowohl jüngere Schwangere (15 bis 19 Jahre) als auch ältere Schwangere (35 bis 45 Jahre) zeigten ein erhöhtes Risiko für einen schweren Verlauf von COVID-19 für Mutter und Kind [19], was auf lediglich eine unserer Patientinnen zutraf. Darüber hinaus wurden eine Co-Infektion mit HIV und eine mütterliche Anämie (Hb-Wert < 11,5 g/dl) zum Zeitpunkt der Diagnosestellung als Risikofaktoren identifiziert [19, 20]. Die Bedeutung der Präeklampsie in diesem Zusammenhang ist unklar [21]. Das Schwangerschaftsstadium zum Zeitpunkt der Infektion scheint kein Risikofaktor zu sein [22]. Insgesamt wird das Risiko eines schweren Verlaufs einer COVID-19-Erkrankung bei schwangeren Frauen im Vergleich zu nichtschwangeren Frauen als höher eingeschätzt [19, 20].

In unserer Kohorte waren Schwangere mit Migrationsgeschichte und Sprachbarrieren überrepräsentiert (7 von 9 Patientinnen). Die Datenlage hierzu ist sehr begrenzt. Der Nachweis, dass Schwangere, die einer bestimmten ethnischen Gruppe angehören, per se ein erhöhtes Risiko für einen schweren Verlauf von COVID-19 haben, konnte bisher nicht erbracht werden. In einer 2023 vom Bundesamt für Migration und Flüchtlinge veröffentlichten Studie waren 2021 Personen mit Migrationserfahrung jedoch fast doppelt so häufig an COVID-19 erkrankt und seltener geimpft [23]. Der Zusammenhang kann sich aus einem schlechteren Zugang zu präventiven Maßnahmen der Gesundheitsversorgung und aus Informationsdefiziten aufgrund von Sprachdefiziten ergeben.

### Impfung und Schwangerschaft

Schwangere haben im Vergleich zu nichtschwangeren Frauen ein erhöhtes Risiko für schwere Verläufe bestimmter Infektionskrankheiten. Dies ist z. T. darauf zurückzuführen, dass das Immunsystem während der Schwangerschaft moduliert wird, um die Toleranz des Fetus zu gewährleisten [24]. Es gibt Hinweise darauf, dass eine COVID-19-Erkrankung während der Schwangerschaft mit einer erhöhten Rate von Komplikationen wie Präeklampsie, Frühgeburtlichkeit und erhöhter mütterlicher Sterblichkeit einhergeht [24]. Ziele der Impfung von Schwangeren sind die Verhütung von Morbidität und Mortalität bei der Mutter und die Übertragung einer passiven Immunität auf das Neugeborene [25]. Impfungen, insbesondere gegen Tetanus, Diphtherie und Keuchhusten, verringern nachweislich die Morbidität und Mortalität von Müttern und Säuglingen [26]. Dagan et al. stellten bei schwangeren Patientinnen eine hohe Wirksamkeit der vollständigen Impfung in Bezug auf die Infektionswahrscheinlichkeit mit SARS-CoV‑2 von ca. 95 % fest [27, 28]. Die Antikörpertiter nach der COVID-19-Impfung sind bei schwangeren und nichtschwangeren Frauen vergleichbar [29].

Antikörper werden sowohl im Nabelschnurblut als auch in der Muttermilch gefunden [29]. Die COVID-19-Impfung wird daher ausdrücklich für alle Frauen im gebärfähigen Alter empfohlen. Für nichtgeimpfte Schwangere wird die Impfung im 2. Trimester empfohlen [28]. Allerdings gibt es derzeit keine randomisierten kontrollierten Studien zur Sicherheit von SARS-CoV-2-Impfstoffen bei Schwangeren. Die Ergebnisse einer großen retrospektiven kanadischen Kohortenstudie mit mehr als 85.000 Patientinnen, die während der Schwangerschaft gegen COVID-19 geimpft wurden, deuten darauf hin, dass die COVID-19-Impfung bei Schwangeren nicht mit einem erhöhten Risiko für Frühgeburt, niedrigem Geburtsgewicht oder Fruchttod verbunden ist [30].

### Radiologische Bildgebung und Strahlenschutz

Acht der 9 Patientinnen erhielten trotz Schwangerschaft eine kontrastmittelgestützte Computertomographie des Thorax. Ionisierende Strahlung kann grundsätzlich Zellen dauerhaft schädigen (Bundesamt für Strahlenschutz: „Informationen für Schwangere“), daher sollte sie im Rahmen der radiologischen Diagnostik nur eingesetzt werden, wenn mit hoher Wahrscheinlichkeit wichtige Erkenntnisse für die weitere Therapie gewonnen werden können und keine geeignete strahlungsfreie oder strahlungsärmere Alternative zur Verfügung steht.

Die Computertomographie des Thorax ermöglicht u. a. die Darstellung von Lungeninfiltraten, Stauungen und Lungenembolien, die bei schweren Verlaufsformen von COVID-19 häufig auftreten und deren genaue Kenntnis für eine optimale Therapieplanung entscheidend sein kann.

Mit einer Energiedosis von ca. 5 mGy [31] liegt die Strahlenexposition bei der modernen Computertomographie deutlich unter der Strahlenexposition, bei der mit einer deterministischen Schädigung des Fetus gerechnet werden muss. Obwohl nach derzeitigem Kenntnisstand in diesen niedrigen Dosisbereichen kein erhöhtes Risiko für Letalität, genetische Schäden, epigenetische Veränderungen, Teratogenität, Wachstumsstörungen oder Sterilität besteht [34], können stochastische Effekte nicht ausgeschlossen werden. Die Entstehung von Leukämien und soliden Tumoren kann bereits in der pränatalen Entwicklung induziert werden und sich erst Jahre später klinisch manifestieren [32]. Korrespondierend dazu befürworten die Leitlinien des American College of Obstetricians and Gynecologists [33] den Einsatz der Computertomographie bei Schwangeren nur unter der Voraussetzung, dass die Untersuchung die notwendigen Informationen liefert und sich daraus therapeutische Konsequenzen ableiten lassen. Dies war nach unserer Einschätzung bei unseren Patientinnen jeweils der Fall.

Ist für eine computertomographische Bildgebung die Gabe eines Kontrastmittels erforderlich, z. B. zum Nachweis einer Lungenembolie, so sollte dies unter Berücksichtigung des individuellen Risikos in möglichst geringer Dosis verabreicht werden. Bei maternaler Kontrastmittelexposition nach der 12. Schwangerschaftswoche sollte die Schilddrüsenfunktion des Neugeborenen innerhalb der ersten Lebenswoche überprüft werden [34].

Grundsätzlich wird die Nephrotoxizität von Kontrastmitteln im klinischen Alltag vermutlich überschätzt: So fanden Obed et al. in einer Metaanalyse kein erhöhtes Risiko für akutes Nierenversagen, Dialysepflichtigkeit oder erhöhte Mortalität durch Kontrastmittelgabe im Rahmen von Computertomographien bei Patienten mit einer eGFR > 45 ml/min/1,73 m^2^KOF [35].

Grundsätzlich stellt die Magnetresonanztomographie (MRT) , die ohne ionisierende Strahlung auskommt, eine diagnostische Alternative zur Computertomographie dar. Nachteile dieser Methode sind u. a. eine längere Untersuchungsdauer und die Tatsache, dass Metalle am und im Körper zu Bildstörungen führen. Für eine MRT-Untersuchung ist ein längeres Liegen erforderlich. Dies kann zu einer weiteren Verschlechterung der respiratorischen Insuffizienz führen, und respiratorische Beatmungshilfen wie nasale Hochflusstherapie oder nichtinvasive Beatmung sind im MRT technisch nicht sicher zu etablieren. Angesichts des logistischen Aufwands und des schwierigen Zugangs zu Patientinnen im MRT haben wir uns bei unseren Patientinnen mit schwerer respiratorischer Insuffizienz bewusst für die Computertomographie entschieden. Die Vor- und Nachteile beider Verfahren sollten jedoch im Einzelfall gegeneinander abgewogen werden.

### Fieber und Schwangerschaft

Bereits 1998 berichteten Chambers et al. über einen teratogenen Effekt von Fieber in der Frühschwangerschaft [38]. Dreier et al. beschrieben ein erhöhtes Risiko für das Auftreten von Neuralrohrdefekten, Herzfehlern und Lippen-Kiefer-Gaumen-Spalten [39]. Andere Autoren erwähnen auch ein erhöhtes Risiko für das Auftreten von atrioventrikulären Septumdefekten, rechtsseitigen obstruktiven Läsionen, Trikuspidalatresie oder Transposition der großen Arterien aufgrund von Fieber während der Schwangerschaft [36]. Wir strebten deshalb eine Körpertemperatur unter 38 °C für unsere Patientinnen an und realisierten das pharmakologisch mit Paracetamol und durch physikalische Maßnahmen wie Wadenwickel, kalte Infusionen und Pfefferminzwaschungen. Bei ECMO-Patientinnen konnte die Temperaturkontrolle über das Extrakorporalsystem gewährleistet werden.

### Schwangerschaft und Bauchlagerungstherapie

Die prolongierte Bauchlagerungstherapie ist ein wesentlicher evidenzbasierter Pfeiler in der Therapie von Patienten mit akutem Atemnotsyndrom [37]. Die Datenlage zur Anwendung der prolongierten Bauchlage bei Schwangeren ist jedoch begrenzt. Unserer Erfahrung nach kann sie auch in fortgeschrittenen Stadien der Schwangerschaft sicher angewendet werden. Die Berücksichtigung des graviden Uterus bei der Lagerung wird empfohlen [38] und kann im Einzelfall eine Herausforderung darstellen. Andernfalls kann es zu einer unzureichenden Durchblutung der Gebärmutter, der Plazenta und des Fetus kommen [38]. Alle Patientinnen wurden täglich von einer Gynäkologin bzw. einem Gynäkologen untersucht, einschließlich der qualifizierten Durchführung einer Dopplersonographie. Nach Indikationsstellung zur Lagerungstherapie (CARDS, p_a_O_2_/F_I_O_2_-Index < 150 mm Hg trotz PEEP von 14 mm Hg) führten wir eine orientierende sonographische Untersuchung des Fetus mit Fokus auf die fetale Herzaktion und Kindsbewegungen durch, um eine intakte Schwangerschaft zu dokumentieren. Diese sonographischen Untersuchungen wurden dann vor und nach jeder 16-stündigen Lagerungstherapie wiederholt. Vor Verbringen in die Bauchlage wurde eine inhalative Sedierung mit Sevofluran begonnen, idealerweise unter Aufrechterhaltung der assistierten Spontanatmung bis zum Erreichen eines RAAS von −4. Mit diesem Vorgehen hatten wir bei der Behandlung von Patientinnen und Patienten mit CARDS gute Erfahrungen gemacht, und das Behandlungsteam war mit der Methode vertraut [39]. Die 16-stündige Bauchlagerungstherapie wurde im direkten Wechsel mit sitzender Position in einem Mobilisationsstuhl (Mobilizer® NORBERT [Reha & Medi Hoffmann GmbH, Leipzig, Deutschland]), der für den Transfer in eine horizontale Position gebracht werden konnte, durchgeführt, sodass eine längere flache Rückenlage weitestgehend vermieden wurde. Für die Durchführung wurde ein Team von mindestens 5 Personen eingesetzt (eine Person am Kopf zur Fixierung des Endotrachealtubus und je 2 Personen seitlich, bei ECMO-Patientinnen zusätzlich eine Person zur Fixierung der ECMO-Kanülen und Überwachung der ECMO-Pumpenflüsse). Zur freien Lagerung des Abdomens wurden Lagerungskissen (20 × 20 × 100 cm) unter Schultergürtel und Becken verwendet. Zusätzlich wurde ein Lagerungskissen aus Schaumstoff mit einer Neigung von 15° verwendet. Eine kontinuierliche Überwachung des Fetus ist wünschenswert, stellt aber auch eine erhebliche personelle und technische Herausforderung dar [38] und ist mit dem Risiko einer Druckschädigung der Mutter durch die an Bauchlage angebrachten Empfängermodule verbunden. Durch eine engmaschige und sorgfältige klinische Überwachung der Schwangeren kann ein Vena-cava-Kompressionssyndrom (Tachykardie, Blutdruckabfall, obere Einflussstauung, plötzlicher ZVD-Anstieg) frühzeitig erkannt und Gegenmaßnahmen können rasch eingeleitet werden. Gleiches gilt für die Lagerung von Schwangeren mit ECMO. Ein im Umgang mit ECMO-Patienten erfahrenes Team, ein adäquater Volumenstatus und ein gutes klinisches Urteilsvermögen sind hier der Schlüssel zum Erfolg.

### Inhalatives Stickstoffmonoxid und Prostazyklin

Bei schwerer respiratorischer Insuffizienz kann eine inhalative Therapie mit Stickstoffmonoxid oder Prostazyklin (Iloprost) durch Optimierung des Verhältnisses von Ventilation und pulmonaler Perfusion zu einer Verbesserung der Oxygenierung beitragen. Beim ARDS wird die grundsätzliche Gabe von inhalativem NO nicht mehr empfohlen, da bei routinemäßiger Anwendung keine Verbesserung der Letalität nachgewiesen werden konnte. Individuell und akut kann jedoch durch die Behandlung mit inhalativem NO eine lebensbedrohliche Hypoxie verhindert werden [40]. Randomisierte Studien zur Sicherheit von iNO bei Schwangeren fehlen. In einer Fallserie mit 6 Patientinnen aus dem Jahr 2020 zeigte die intermittierende Behandlung mit iNO bei Schwangeren eine vorübergehende Verbesserung der systemischen Oxygenierung und eine gute klinische Verträglichkeit. Haupteffekte von iNO sind eine selektive pulmonale Vasodilatation und eine leichte Dilatation der Bronchien [41].

Kleinere Fallserien und Fallberichte haben gezeigt, dass die Anwendung von Iloprost in der Schwangerschaft nicht mit einer erhöhten mütterlichen oder fetalen Mortalität assoziiert ist [42, 43].

### ECMO, Kanülierung und Auswirkungen auf das ungeborene Kind

Die Kompression der V. cava inferior durch den Uterus kann zu Problemen bei der Einführung von ECMO-Kanülen über die Femoralvenen führen und die Flusseigenschaften negativ beeinflussen. Einige Autoren empfehlen daher die Verwendung von Doppellumenkanülen, die über die rechte V. jugularis interna eingeführt werden [44]. Andere Autoren beschreiben diese Probleme nicht und empfehlen die bifemorale Kanülierung [45]. Für uns waren individuelle anatomische Verhältnisse ausschlaggebend. Da wir ausschließlich armierte Kanülen verwenden und diese in jedem Fall bis nach intrathorakal einführen, sind Kompressionsphänomene durch den Uterus unserer Ansicht nach zu vernachlässigen. Es wurden sowohl doppellumige bikavale Kanülen mit 27 F als auch transfemorale Kanülen mit 21 F verwendet. Beide Techniken funktionierten zuverlässig. Die Punktion der Venen bzw. die Platzierung der Kanülen im rechten Vorhof erfolgte jeweils unter echokardiographischer Kontrolle. Die Anwendung einer venovenösen ECMO-Therapie ist keine ARDS-Therapie im eigentlichen Sinne. Es handelt sich ausschließlich um ein lebenserhaltendes Verfahren. Der Einsatz der ECMO dient lediglich dem Zeitgewinn für eine mortalitätssenkende Lagerungstherapie. Die häufigen Bauchlagerungen (je 12 Bauchlagerungen bei beiden ECMO-Patientinnen) erforderten regelmäßige sonographische Lagekontrollen mit Korrekturen der Kanülenposition. Leider kam es bei beiden ECMO-Patientinnen zum intrauterinen Fruchttod. Wir vermuten hier am ehesten eine plazentare Thrombose durch die COVID 19-bedingte Thrombophilie dieser Patientinnen [46]. In der Literatur werden hohe fetale Überlebensraten nach ECMO-Therapie berichtet [47]. Die häufigsten ECMO-bedingten Komplikationen sind Blutungskomplikationen, Embolien und bakterielle Superinfektionen [48]. Ein Protokoll zur Überwachung der Blutgerinnung kann die Häufigkeit von Blutungskomplikationen und Embolien verringern [49]. Die bisher größte Multizenterstudie [50] zeigte ermutigende Überlebensraten (Mütter: 76 %, 59 % lebende Neugeborene), aber auch lange intensivmedizinische Behandlungsbedürftigkeit und hohe Komplikationsraten.

### Maternale Hyperkapnie

Das Zulassen einer reduzierten Ventilation (permissive Hyperkapnie) zur Reduktion der Beatmungsinvasivität ist ein etabliertes Konzept im Rahmen einer „lungenprotektiven Beatmung“ [51]. Bei Schwangeren ist dieser Ansatz jedoch problematisch. Die mit der Schwangerschaft einhergehende erhöhte Ventilation führt zu einer metabolisch kompensierten respiratorischen Alkalose mit mütterlichen CO_2_-Werten von ~30 mm Hg. Der effektive CO_2_-Transfer vom fetalen in den mütterlichen Kreislauf ist von einem Diffusionsgradienten von ca. 10 mm Hg abhängig [52].

Ein fehlender Diffusionsgradient führt zu einem unzureichenden oder fehlenden CO_2_-Tranfer vom Ungeborenen zur Mutter und im ungünstigsten Fall sogar zu einer Diffusionsumkehr. Mütterliche Hyperkapnie kann so zu fetaler Hyperkapnie und Acidose führen [53].

Eine Acidose verschiebt die fetale Sauerstoffdissoziationskurve nach rechts, wodurch die Fähigkeit des fetalen Hämoglobins, Sauerstoff zu binden, verringert wird [52]. Als bedeutender Risikofaktor für neonatale Morbidität und spätere neurologische Schäden sollte eine maternale Acidose daher konsequent verhindert werden.

Kann trotz Anpassung der Beatmungsparameter, wie z. B. eine Erhöhung der Atemfrequenz, keine Normokapnie erreicht werden, kann die Implantation einer vv-ECMO eine Therapieoption darstellen [50]. Neben der Sicherstellung der Oxygenierung bietet die vv-ECMO die Möglichkeit der Normoventilation bei gleichzeitiger Reduktion der Invasivität der maschinellen Beatmung.

### Optimaler Geburtstermin

Es besteht ein breiter Konsens darüber, dass eine COVID-19-Erkrankung allein keine Indikation für eine Entbindung darstellt [54]. Grundsätzlich sollte, wenn es der Zustand der Mutter zulässt, eine Entbindung zum errechneten Geburtstermin angestrebt werden. Wenn im Rahmen der Therapie noch keine Kortikosteroide verabreicht wurden, kann bei drohender Frühgeburtlichkeit eine antenatale Steroidgabe („Lungenreife“) nach geburtshilflichen Kriterien bis zur 34 + 0 SSW mit Betamethason bzw. Dexamethason erfolgen [55]. Bei der kritisch kranken geburtshilflichen Patientin mit COVID-19 sollte in enger interdisziplinärer Abstimmung zwischen Gynäkologen, Neonatologen und Intensivmedizinern zwischen einer weiteren COVID-19-Therapie mit Prolongation der Schwangerschaft und einer Geburtseinleitung abgewogen werden [12]. Grundsätzlich ist die Anwesenheit einer gesunden Begleitperson bei der Geburt möglich und sinnvoll [54].

### Geburtshilfliche Anästhesie

Bei gesunden Schwangeren empfiehlt die aktuelle S1-Leitlinie der Deutschen Gesellschaft für Anästhesiologie und Intensivmedizin die Spinalanästhesie bei Fehlen von Kontraindikationen für den Kaiserschnitt als Verfahren der Wahl, weist aber auch darauf hin, dass die Allgemeinanästhesie insbesondere bei Notfallindikationen das geeignetste Anästhesieverfahren für die Kaiserschnittentbindung sein kann. Bei erhöhtem Aspirationsrisiko wird eine „rapid sequence induction“ (RSI) zur Narkoseeinleitung empfohlen [56]. Bei der speziellen Gruppe der Patientinnen mit CARDS ist bei leichteren Verlaufsformen eine Kaiserschnittentbindung in Regionalanästhesie denkbar und die Vermeidung einer Intubation bei einer bisher nichtintubierten Patientin ein erstrebenswertes Ziel.

Die bei diesen Patientinnen durchgeführte therapeutische Antikoagulation muss rechtzeitig beendet werden. Ob die Gebärende eine Flachlagerung für die Dauer der Sectio caesarea toleriert, sollte im Vorfeld kritisch abgewogen und, wenn möglich, mit der Patientin besprochen und außerhalb des Kreißsaals ausprobiert werden. Die Anästhesie sollte von einem in geburtshilflicher Anästhesie erfahrenen Anästhesisten durchgeführt werden. Mögliche Vorteile der Spinalanästhesie gegenüber der Allgemeinanästhesie sind eine gute perioperative Schmerzlinderung, die Möglichkeit der Anwesenheit einer Begleitperson, ein früherer Haut-zu-Haut-Kontakt mit dem Neugeborenen, die Vermeidung einer möglicherweise schwierigen Intubation, ein geringerer Blutverlust und weniger Aerosole durch Manipulationen an den Atemwegen [57]. Der Schutz und die Sicherheit des Behandlungsteams sollten durch geeignete Behandlungsprotokolle sichergestellt werden.

Bei Patientinnen mit schweren Verlaufsformen von CARDS, insbesondere wenn bereits eine endotracheale Intubation besteht, ist die Allgemeinanästhesie das Verfahren der Wahl [57].

## Fazit für die Praxis

- Hohe Überlebensraten bei schwangeren Patientinnen mit CARDS sind möglich. Eckpfeiler der Behandlung von Schwangeren mit CARDS sind die Bauchlagerungstherapie mit druckreduzierter Lagerung des Uterus, die Vermeidung von Hyperkapnie zur Verhinderung negativer Auswirkungen auf die uterine Perfusion, die Fiebervermeidung und die Wahl des optimalen Zeitpunktes für Geburtseinleitung oder Sectio caesarea in enger interdisziplinärer Absprache.
- Wenn eine extrakorporale Membranoxygenierung indiziert ist, sollte die Kanülierung an die individuellen anatomischen Bedingungen angepasst werden. Die transjuguläre Doppellumenkanüle über die V. jugularis interna kann von Vorteil sein, falls eine Flussbehinderung der femoralen Gefäße durch die Schwangerschaft befürchtet wird.
- Für eine Sectio caesarea kann bei Patientinnen mit mildem CARDS bei guter Planung eine neuroaxiale Anästhesie sicher durchgeführt werden. Die für COVID-19 empfohlene therapeutische Antikoagulation kann jedoch das Risiko von Blutungskomplikationen erhöhen, sodass eine Allgemeinanästhesie insbesondere bei schweren Verläufen die praktikablere Alternative darstellt.
